# Brunn: An open source laboratory information system for microplates with a graphical plate layout design process

**DOI:** 10.1186/1471-2105-12-179

**Published:** 2011-05-20

**Authors:** Jonathan Alvarsson, Claes Andersson, Ola Spjuth, Rolf Larsson, Jarl ES Wikberg

**Affiliations:** 1 Department of Pharmaceutical Biosciences, Uppsala University, Uppsala, Sweden; 2 Department of Medical Sciences, Uppsala University, Uppsala University Hospital, Uppsala, Sweden

## Abstract

**Background:**

Compound profiling and drug screening generates large amounts of data and is generally based on microplate assays. Current information systems used for handling this are mainly commercial, closed source, expensive, and heavyweight and there is a need for a flexible lightweight open system for handling plate design, and validation and preparation of data.

**Results:**

A Bioclipse plugin consisting of a client part and a relational database was constructed. A multiple-step plate layout point-and-click interface was implemented inside Bioclipse. The system contains a data validation step, where outliers can be removed, and finally a plate report with all relevant calculated data, including dose-response curves.

**Conclusions:**

Brunn is capable of handling the data from microplate assays. It can create dose-response curves and calculate IC_50 _values. Using a system of this sort facilitates work in the laboratory. Being able to reuse already constructed plates and plate layouts by starting out from an earlier step in the plate layout design process saves time and cuts down on error sources.

## Background

The life sciences of today is an information-heavy field with extensive use of high-throughput methods that generate large amounts of data at an ever-increasing rate [1,2]. As the quantity of data increases, proper methods for handling the data become important. Good data handling with standardization is important not only in the analysis step, but also for the data generation step [3]. Modeling wet-laboratory experiments in a computerized information system is a non-trivial task and needs a flexible system capable of supporting the daily laborative work related to microplates, which are the main media used in high-throughput assay systems.

General systems for ultra high throughput screening need highly optimized and streamlined platforms [4], whereas lead optimization systems need to be more flexible and handle a more fluctuating range of plate formats and layouts [5]. This study was devoted to the development of such a flexible laboratory information system (LIS) for microplates, termed "Brunn". Bioclipse [6] was choosen as a platform to base the LIS on. The system is designed to help in refining raw data and encourages re-use of layouts for the plates, Brunn thus focuses on providing a platform for fluctuating plate layouts; here we demonstrate it on a fluorometric microculture cytotoxicity assay (FMCA) in use at the university hospital of Uppsala, Sweden.

### Microplates

Microplates were introduced in the early 1960s [7]. Since then they have become an integral part of daily laboratory work in most disciplines, including drug discovery and clinical routine testing. Microplates consist of an array of wells. The outer dimension of microplates is standardized, but the plates are available with different numbers of wells and volumes. Microplates can be handled both manually and by robots, and they facilitate sample handling and serial dilutions by transferring solutions across rows or columns in parallel. They can be manufactured with different optical properties, such as for fluorescent read-outs, colorimetric assays, and microscopy. In addition, the plastics can be given different properties, *e.g*. making a sample adhere, or not adhere, to the walls of the well. Moreover, wells can be shaped in different ways (most common are flat and V-shaped bottoms). Microplates are available in a variety of standardized densities, where 8 rows × 12 columns = 96 wells with a volume of 200-300 *μL *are the most suited to be handled manually. Much denser plates are also available, among which 384-well plates can be handled manually, though this is rather painstaking. Plates with higher densities than this are difficult to use; one reason for this is that the small volumes of liquid in each well evaporate quite fast.

Microplates allow large quantities of data to be generated fast. Most assays give one result per well, but it is also possible to perform time-resolved assays with one measurement per time-point per well. In high-throughput settings, thousands of measurements are generated by automatic plate handling robots, of which many types are available on the market. Collecting high-throughput data in a laboratory information system gives great benefits. For example, assays commonly exhibit a systematic variation across the geometry of the plate, which necessitates controls to be distributed in a statistically secured way over the plates. In order to handle this effectively a LIS for microplates is required.

### Bioclipse

The first version of Bioclipse was released in 2005 and Bioclipse has since then been in constant dynamic development and improvement. Bioclipse is an open source software integration platform for the life sciences, providing a graphical point-and-click workbench for chemo- and bioinformatics data. Bioclipse features a plug-in-based architecture which makes its possible to seamlessly integrate virtually any functionality in Bioclipse and make it function with any other Bioclipse functionality. Among its functions can be mentioned QSAR functionality [8], site-of-metabolism prediction [9], semantic web support [10], remote web services [11], browsing of large compound collections, editing of chemical structures, and a reporting system based on JasperReports [12], which can be used for generating printable reports with text and diagrams. On top of this, a very powerful feature of Bioclipse is also its JavaScript environment through which the entire application can be scripted [13].

Bioclipse inherits the top-of-the-line plugin structure of Eclipse, which provides an extensible environment where each Bioclipse installation can be tailored with the features needed by a certain user. Choosing Bioclipse as the platform for Brunn means that Brunn does not need to reinvent the proverbial wheel, but can use suitable parts of Bioclipse already available, and it allows for the easy integration of the microplate assay data with the other features of Bioclipse. The Bioclipse workbench consists of editors and views. Editors follow the load/save pattern and many instances of the same editor can be open at the same time, whereas views reside around the editors and shows different information; for example a table of properties for the selected resource.

## Implementation

### Architecture

Brunn ships as a set of plugins for Bioclipse. For example, a core plugin contains the business logic, and a user interface plugin contains the graphical visualisation parts.

**Figure 1:**
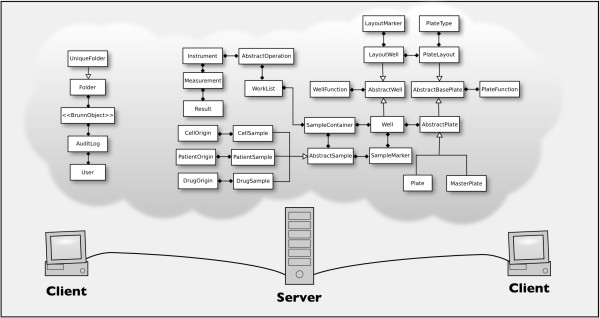
**Architecture**. Brunn is a client-server system. The program runs on the client, whereas the server hosts the database. The data model reflects the stepwise way in which plates are constructed in Brunn. For example, during creation of a MasterPlate, the LayoutWells and LayoutMarkers are used as templates for creating the Wells and SampleMarkers. (Please note that the figure depicts a simplified version of Brunn's data model. The complete version is available at the Brunn webpage [27].)

A simplified version of the data model is shown in Figure 1. The relational database stores measurements in a general way, and each well has a queue of operations that can be performed on it. Brunn currently uses only one standard measurement operation, but with the elected architecture it is relatively easy to adopt Brunn to a workflow where multiple measurements or operations are performed on each well. The data model contains a base entity AbstractSample, which can be built upon for creating samples other than the currently supported ones.

Brunn uses Hibernate [14] for mapping objects to the relational database, which is implemented in MySQL [15], and the Spring Framework [16] to leverage communication with MySQL.

### User Interface

**Figure 2:**
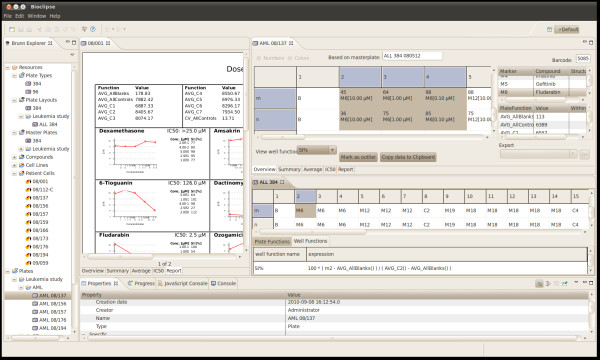
**Screenshot from Brunn**. Screenshot from a running Brunn client. To the left is the Brunn explorer tree view, containing all Brunn resources. At the bottom is the properties view, showing properties for the selected Plate in Brunn explorer. Directly to the right of the Brunn Explorer is the plate report tab of a Plate editor with graphs and calculated IC_50_-values. To the top right is shown another Plate editor; this one shows the Overview tab and below that is shown a PlateLayout editor, showing the well function for SI % on a well.

Brunn's user interface consists mainly of the *Brunn Explorer *- a tree view for browsing resources - and a number of editors used for editing and visualising them (Figure 2). Computer representations of plates are created using an iterative process where each step is stored as one resource type. Table 1 contains a complete list of Brunn resources visible in the Brunn Explorer.

**Table 1:** Resources in Brunn

PlateType	A PlateType defines the size of a plate.
PlateLayout	A PlateLayout defines which wells are used for dilution series, blank and controls. It also defines all calculation functions.
MasterPlate	A MasterPlate defines which substances with which concentrations are in which wells.
Compound	A Compound is a substance whose effects are of interest.
Cell Line	A Cell Line represents a cultured cell type that is grown outside the body. Can be bred for specific properties such as drug resistance
Patient Cell	A Patient Cell represents tissue samples from a patient.
Plate	A Plate corresponds to a real life plate and is what result data is coupled to.

Plates in Brunn are built in a stepwise process where the result of each step is stored in the database as one resource type.

## Results

**Figure 3:**
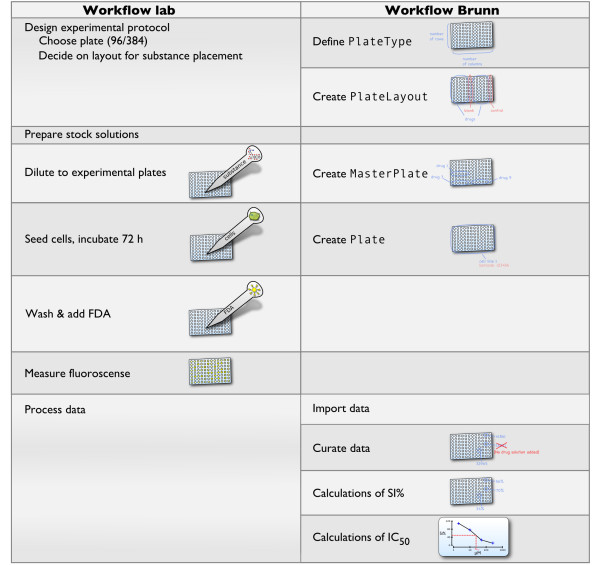
**Comparison of the workflows in the wet-lab and in Brunn**. The left column shows a schematic representation of what is done in the wet-lab (*cf*. the FMCA protocol publication [21]) and the right column shows the corresponding operations in Brunn.

Brunn is modeled around the workflow exemplified in the left part of Figure 3 which shows an example use case for Brunn. PlateTypes of any size can be defined by giving the number of rows and columns. (Some parts of Brunn currently only support 96- and 384-well plates placing a restriction on this, however). When creating a PlateLayout, the user defines which wells should be used as controls, and which for treatments. In this step, Excel-like formulæ can be entered for calculating, for example, survival index in percent (SI %). Substances are placed on the plates in batches. All plates in a batch conform to a MasterPlate defining which substances are in which wells and in which concentrations. When a plate is to be used, it is retrieved from cryostorage and samples are seeded on it and assay specific steps are taken to retrieve raw data which are imported into Brunn. Imported data can be curated manually. Values such as SI % can be calculated using values from the wells and the formulæ defined when creating the PlateLayout.

Dose-response curves can also automatically be generated and IC_50_-values calculated.

### Auditing and Users

Brunn has two user levels, administrators and regular users. An administrator can do what all regular users can do, as well as create new users and undelete resources which previously have been deleted. Brunn keeps track of who created what resource and when. When a resource is selected in the Brunn Explorer, the Properties View will list creator and creation date for that resource, as shown in Figure 2. After a resource has been created in Brunn, for security reasons, it can actually not be deleted - only marked as deleted.

### Defining a PlateType

It is possible to create Plates of any size by defining a PlateType with the desired number of rows and columns. There are no restrictions in the data model for the sizes of the plates, but as mentioned above currently not all parts of Brunn support sizes other than 96- and 384-well microplates.

### Creating a PlateLayout

When working with microtiter plates it is common to use the same general layout and calculate the same values for the same wells for multiple plates. In order for the user to only have to input the information once, a reusable PlateLayout can be created. PlateLayouts are created from PlateTypes. The PlateLayout editor has a point-and-click interface for placing well markers symbolizing different groups of control, blank and treatment wells. When control markers are placed, functions for average and standard deviation for the wells with those markers are automatically created. The PlateLayout editor also contains a user interface for creating other user-defined plate-specific functions.

In order to compensate for drift within the plate, different control wells can be used when calculating things like survival index for various wells. This means that well-functions, which are containers for well-specific calculation formulæ, for example SI %, need to be created manually only once for each group of wells using the same control.

Well functions and Plate functions make it possible for the user to create a PlateLayout, tailored to calculate exactly what the user is intrested in; for example survival index or various statistical measures used during primary screening. (See Figure 2 for an example of a Well function.)

### Placing substances - defining a MasterPlate

Plates are normally prepared and cryostored in batches where all plates in one batch have the same substance with the same concentration at the same location. In a subsequent step, they are seeded with samples. Creating such a batch of plates corresponds to creating a MasterPlate in Brunn.

In the MasterPlate editor, the substance markers placed in the PlateLayout editor are associated with a substance with a different concentration for each well. This is done by dragging-and-dropping a substance to a marker and creating a dilution series in a dialog window.

### Adding samples - creating a Plate

The seeding of a physical plate with samples corresponds to creating a Plate in Brunn. The entity Plate corresponds to a microplate with a barcode, substances, and samples seeded on it. The dialog window for creating a Plate lets the user specify barcode, plate name, cells to seed and a MasterPlate to base the new Plate on. Once a plate has been created, data can be imported to it.

### Data importing

**Figure 4:**
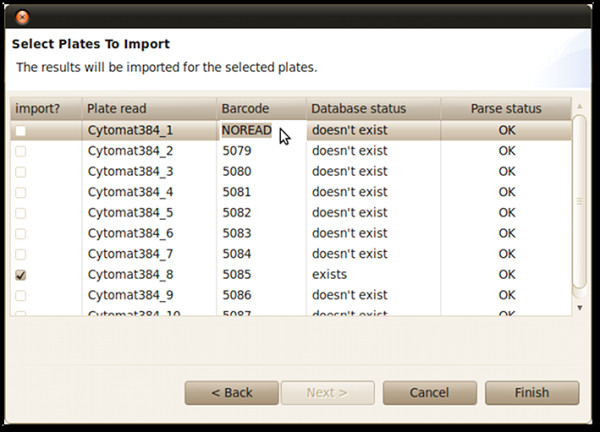
**Data import dialog**. Shown is the data import dialog for importing a batch of 384 well plates, read from the FLUOstar Optima. If the barcode reader has failed, or if a file format is read that does not contain barcodes, the barcode field has to be entered manually, based on the order of the plate run. The system shows whether or not a plate with the corresponding barcode exists in the database. If such a plate exists the user can select to import the data for it.

Results are imported into Brunn from a text file containing raw data from one or many plates. Brunn supports imports from two different file formats, namely data log files from the multipurpose reader FLUOstar Optima (BMG Labtech) and plain comma separated files. Files produced by an automated system may already contain barcodes, but in case the file lacks a certain barcode, it must be provided during the import step as shown in Figure 4. Raw data is connected to Plates in Brunn matched by barcodes.

### Data browsing and curation

Once raw data has been imported, the Plate editor can be used for viewing the results for one Plate. The Plate editor has multiple tabs where data can be viewed in different ways (see Figure 2).

By using the proper tabs of the Plate editor, wells can be marked as outliers, effectively removing them from further calculations. To help the user identify outliers the calculation functions can be viewed. For example, when a plate layout is created with markers indicating wells used for blank and control values, functions like coefficient of variance as percent (CV %) for all wells with those markers are automaticly created, or if doing primary screening the user can write a plate function for standard deviation of all screened substances and for example use it when creating well functions, like Z-scores [17].

The last tab in the Plate editor shows a graphical report containing dose-response curves and automatic calculations of IC_50_. The dose response curves are drawn with straight lines between the points and the IC_50 _values are calculated by identifying the two points closest to 50% and then doing a linear aproximation between them to get a value for IC_50_. This is the default behavior of Brunn because when working with cell assays the shape of the curves tend to vary a lot. However, if the assay gives output suitable for a sigmoidal curve fitting there is a user configurable preference that toggles IC_50_-calculation by fitting the Hill equation using non-linear least-squares regression with the org.apache.commons.math [18] library.

### Accessing the Brunn database in Bioclipse

The data in the Brunn database can be reached via the Bioclipse interface through the scripting language JavaScript. This means that more advanced analyses based on Brunn data can be performed by a user familiar with this tool. By retrieving data from the database, data format consistency is guaranteed regardless of who made the actual lab work.

**Figure 5:**
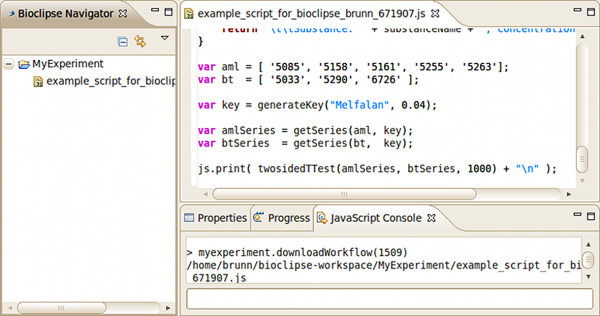
**A JavaScript example**. The figure shows a part of an example JavaScript script opened in the JavaScript editor. The visible rows of the script defines two lists of barcodes and a key for a certain concentration of Melfalan. It then calls functions defined earlier for retrieving the data series from the database and for performing a t-test on the data. Notice also how the Bioclipse funtionality for downloading a JavaScript script from *^my^*Experiment by the JavaScript console in Bioclipse has been used.

An example of a JavaScript script [19] performing a double-sided t-test on Brunn data can be found at the *^my^*Experiment [20] web page. (See also Figure 5.)

### Built-in documentation

Bioclipse provides a built-in help system where the user can either browse -- much like one can browse the web -- or have a help view open which shows context-specific assistance for what the user is doing for the moment. The documentation system also features small stepwise instructions called cheat-sheets for common operations. Brunn takes advantage of both these features. Table 2 lists the cheat-sheets currently shipping with Brunn.

**Table 2:** Cheat Sheets in Brunn

**Creating a **PlateLayout	Shows how a PlateLayout with layout markers and calculation functions is created.
**Creating a **MasterPlate	Shows how a MasterPlate is created and how substances are created and added to the MasterPlate.
**Creating a **Plate **and Importing Results**	Shows how a Plate is created, data imported for it and how the results can be viewed.

A cheat-sheet is a stepwise series of instructions inside the program which can help the user remember how certain tasks are performed, or function as a tutorial.

### Example use case: FMCA

While Brunn was developed to be general enough to handle virtually any type of microplate assay, it was validated on a microplate-based fluorometric microculture cytotoxicity assay (FMCA) [21], an assay type that is in wide use for measuring cytotoxic and cytostatic effects of chemical compounds *in vitro*. It is a cell viability assay, and the specific one used herein is in use at the division of Clinical Pharmacology of the Uppsala University Hospital.

Figure 3 shows an overview of the process. Cells are seeded onto 96- or 384-well microplates with dilution series of substances. By measuring fluorescence generated from cells with intact cell membranes, a survival index (SI %) is calculated for each concentration of each substance. In order to accomplish this, some wells on the plate must act as blanks and controls. Moreover, to compensate for systematic variations along the plate, different wells are used as controls for different parts of the plate.

There is a trade-off between testing as large a number of substances as possible on one plate with enough number of replicates, and testing at a sufficient number of different concentrations. Hence, the layout of the plates (*i.e*. which wells are used for dilution series, and which wells are used for more technical ends such as controls and blanks) is continually improved, and there are multiple layouts being used side by side.

## Discussion

### Control markers and Brunn

When working with microplates it is common to use control markers to measure systematic errors. There are statistical methods that complements this sort of markers during primary screening, like for example Z-scores, which can be added as well functions in Brunn, but when doing dilution series they are not applicable. Brideau *et al *[17] lists the following four potential problems with the control based approach:

1. Does not handle positional variability

2. Biases may be added by the controls

3. Control variability is not accounted for

4. Not clear how to handle outliers

Brunn uses built in support for control markers for catching systematic errors and at least partly provides solutions for dealing with these potential problems. In the FMCA example the position variability problem (1) has been approached in two ways. First, there has been trouble with different values in the outermost wells on the plates and hence they have not been used for measurements. Second, there are different control markers on the plate and the closest one is used in the calculations. To keep track of control variability (3) the CV % is calculated both for each control group individually and all controls together.

These CV values can be used as a hint for identifying outliers (4). Still the outlier problem is always present and Brunn does not provide the final solution for how to handle outliers. It is important for the user to have issues like this in mind when designing the platelayout. Brunn provides a framework where things can be set up and organized in a systematic way.

### Comparison of Brunn with other open source LIS

There are two other open source LIS systems for microplate assays which deserve to be mentioned, namely SLIMS [22] and Screensaver [23]. SLIMS is dedicated for chemical genetics and handles large-scale screening data. Screensaver also handles high-throughput screening facilities. However, SLIMS and Screensaver lacks the flexibility of Brunn, as the two former are mainly directed towards primary screening where substances are tested in one concentration to find hits or misses according to some threshold value. When doing mechanistic classifications [24], multiple concentrations need to be assayed and the half maximal inhibitory concentration (IC_50_) calculated from the accumulated data. In the latter situation, which is the situation Brunn is designed for, the goal is rather to learn more about fewer substances. Moreover, the integration of Brunn with Bioclipse allows the experienced Bioclipse user to seamlessly integrate the Brunn features with a wide plethora of ever growing Bioclipse chemobioinformatics resources and plugins.

## Conclusions

Brunn is an open source laboratory information system for microplate based high-throughput screening, capable of handling a multitude of different plate layouts. Brunn can be used to create dose-response curves and extract IC_50_-values. Brunn uses a point-and-click interface based on the graphical workbench Bioclipse and is easy to use and allows integrating data with the multitude of other resources available in Bioclipse.

Brunn ensures that researchers can retrieve data in a consistent manner regardless of who performed the actual lab work. By constructing platelayouts errors from copying and pasting in spreadsheets can be avoided. The more advanced user can extract Brunn data using the Bioclipse scripting environment.

Although the current implementation is very much tailored for the daily work at the division of Clinical Pharmacology at Uppsala University Hospital, the application contains many more general solutions that allow it to be adopted and reused at other locations. The plugin architecture of Bioclipse provides a great flexibility and enables reuse of components built by other Bioclipse projects. It also makes it easy to build plugins for integrating Brunn with other Bioclipse features.

Basing Brunn on Bioclipse has directly brought in a help system including such as cheat-sheets, and scripting possibilities as well as access to a reporting framework (JasperReports). This means also that it will be straightforward for the experienced user to take advantage of the chemobioinformatics tools in Bioclipse.

## Availability and Requirements

**Project name: **Brunn

**Project home page: **http://brunn.sourceforge.net

**Operating system(s): **Platform independent

**Programming language: **Java

**License: **Eclipse Public License (EPL)

**Restrictions to use by non-academics: **none

**Code: **http://github.com/jonalv/bioclipse.brunn

An Oracle VirtualBox image [25] based on Ubuntu with complete installation and some test data can be downloaded from http://pele.farmbio.uu.se/trybrunn/BrunnOnUbuntu.zip

## Authors' contributions

RL and CA conceived the system. JA, CA and OS designed it. JA implemented it, and RL, CA, OS and JW supervised the project. JA, CA, OS and JW wrote the manuscript. All authors read and approved the final manuscript.
